# Looking good but tweeting bad? The social perception of orthodontic-related posts on Twitter and Instagram

**DOI:** 10.1186/s13005-021-00302-1

**Published:** 2022-02-17

**Authors:** Isabelle Graf, Teresa Kruse, Bert Braumann, Karolin Hoefer, Daniel Ehlebracht

**Affiliations:** 1 Department of Orthodontics, Faculty of Medicine and University Hospital of Cologne, University of Cologne, Cologne, Germany; 2 Department of Operative Dentistry and Periodontology, Faculty of Medicine and University Hospital of Cologne, University of Cologne, Cologne, Germany; 3 Institute of Sociology and Social Psychology, Faculty of Management, Economics and Social Sciences, University of Cologne, Cologne, Germany

**Keywords:** Social media, Twitter©, Instagram©, Orthodontics, SAM

## Abstract

**Background:**

Social media plays a major role in the daily life of adolescents and has become highly interesting for healthcare research as well. The aim of this study was to explore the social perception of orthodontic-related posts on Twitter and Instagram by young adults.

**Methods:**

401 orthodontic-related posts were collected during a 30-day period and categorized with regard to specific characteristics – their content and the social networking site (SNS) being used as well as the presence or absence of a *selfie*. In order to investigate the social perception of these posts, 42 young adults rated the emotional states of the SNS users using the Self-Assessment Manikin (SAM)-Tool. A total of 4211 poster-rater observations pertaining to the three SAMs dimensions pleasure, arousal and dominance were analyzed by using linear and multinomial logistic regression analyses.

**Results:**

The investigated characteristics of the collected posts had significant effects on the perceived emotional state of the SNS users. Besides significant SNS-associated differences, there were also effects that were independent of the SNS being used: Receiving orthodontic appliances was more often associated with rather negative emotions (*p* < 0.001), while users who posted about the removal of such braces were more often perceived as joyful (*p* < 0.001). Interestingly, users whose posts contained selfies *with* visible braces were perceived as significantly more positive and stronger in comparison to users who did not post a picture of themselves (*p* < 0.05).

**Conclusion:**

This research gives insights into the social perception of orthodontic-related posts on SNS. While users’ emotional states were perceived highly differential on both SNS, orthodontic-related content also revealed significant effects on social perception. Because selfies with visible braces were associated with positive feelings by young adults, a modern and SNS-related way of coping with a temporary supposed impairment like fixed orthodontic appliances might have been revealed through this research.

**Supplementary Information:**

The online version contains supplementary material available at 10.1186/s13005-021-00302-1.

## Background

Social networking sites (SNS) have become increasingly important in daily life, especially for young adults [1]. Instagram and Twitter are two examples of highly frequented SNS. To take pictures and post them on the *photo*-based platform Instagram seems to be a routine procedure for many adolescents and young adults. Instagram, founded in 2010, is one of the largest SNS worldwide with one billion users [2]. In contrast to Instagram, the SNS Twitter, founded in 2006, is a *text*-based platform where the currently 330 million users can post up to 280 characters and can as well add a picture and/or video [3]. Motives for the use of SNS are social interactions as well as self-expression, among others, depending on the user’s psychological traits [4]. In the context of a social trend towards high esthetics, facial appearance plays a major role for social interactions, communication and self-esteem [5–8]. Interestingly, depressive symptoms in relation to excessive use of SNS were found to be more frequent among Twitter users than among users of other SNS like Facebook [9]. Posts on SNS might cause a reaction by other users in terms of a comment on the respective post and/or a ‘like’ which, as a consequence, might lead to affect the person who initially posted. In relevant literature, the power of other users’ reactions to posts such as ‘likes’ has been stressed, because for some users, ‘likes’ are regarded as rewards or signs of social approval, recognition and support. A high self-esteem seems to be a protective factor for potential harm revolving around the influence of reactions of others such as ‘likes’ [10]. Being Oxford dictionary’s “word of the year” in 2013 [11], a *selfie* – meaning a picture taken of the users showing themselves – can be seen as another social media phenomenon which seems particularly important when looking at posts on the *photo*-based platform Instagram. Researchers regard selfie-taking as a multidimensional phenomenon with approval, belonging and documentation being the key reasons for their use [12, 13]. Thus, social perception is a key factor within researching SNS.

**Fig. 1:**
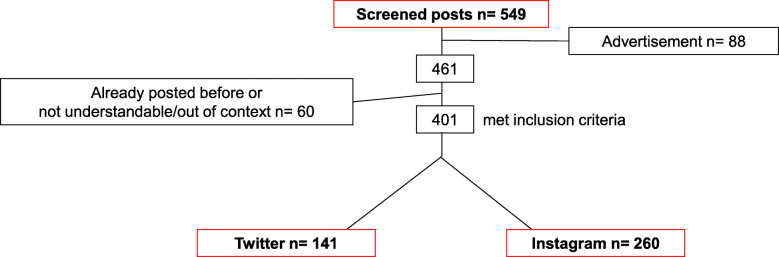
Flow chart of included and excluded posts

Social perception and the underlying emotional dimensions can be investigated through numerous ways, one of them being the use of Self-Assessment Manikins (SAM) [14]. This non-verbal tool rates the three dimensions ‘pleasure’, ‘arousal’ and ‘dominance’ on a five-point pictographic-scale, although seven- and nine-point scales also exist (Fig. 1). Yet, one has to keep in mind that measuring the *perceived* emotional states of subjects might not truly represent the *real* emotions of these subjects, but can be regarded as an important tool for investigating emotional states retrospectively.

In general, SNS do not only play a role in everyday-life, but also in health care and in specific, in the field of orthodontics. Orthodontic patients are mostly adolescents and young adults who are known for their intense and growing interest in the internet and SNS, especially with omnipresent smartphones and the respective possibilities of real-time communication. While some users’ intention is to present pictures of themselves with newly inserted or freshly removed braces, for example, others seek for information about treatment options and help with their current treatment [15]. Whether young patients seek medical advice and/or are willing to undergo orthodontic treatments might crucially depend on the way they subjectively perceive the experiences of others who underwent these treatments – with social media representing an easily accessible source of such information. „*If men define situations as real, they are real in their consequences*” – the so called Thomas Theorem can also be applied within this specific research [16]. Hence, even more than young patients’ actual feelings, which are by definition hidden to the observer, observers’ subjective perception of others’ feelings might be guiding their actions. In addition, SNS have the potential to strengthen the bond between patient and professional through the opportunity to provide information and support [17]. Yet, they are also used by patients to complain about professionals anonymously. The above-mentioned different concepts of the SNS Twitter and Instagram – text-based vs. photo-based – might lead to specific user behavior, being that orthodontic-related posts on Instagram might be seen as more positive than those on Twitter [15]. While the content of orthodontic-related posts has been investigated before [15, 18–22], social perception of such posts, especially with regard to the perceived emotional states of SNS users, have not been dealt with by researches so far. Therefore, the aim of this study was to investigate the social perception of orthodontic-related posts on Twitter and Instagram by young adults.

In specific, we had the following research questions:

- Do young adults perceive the emotional states of SNS users differently based on the content of their posts?
- What role do selfies play in orthodontics and the corresponding social perception?
- Are posts on Twitter rated differently by young adults than posts on Instagram?

## Methods

In the course of a pilot study [15], a previously tested search strategy was applied. This had been done in accordance with relevant literature [21]. Frequently used keywords had been identified as “braces”, “orthodontics” and “orthodontist”. Thus, posts containing one or more keyword within a thirty-day-period of data acquisition were included. Note that only German posts were included. Exclusion criteria were: (a) foreign language posts, (b) advertisement, (c) re-posted material and (d) posts which were not comprehensible [15]. As a result of the above-mentioned, 401 orthodontic-related posts of the SNS Instagram (*n* = 260) and Twitter (*n* = 141) were used for measuring the social perception within the current study (Fig. 1).

All posts were categorized according to their platform (1 = Instagram, 2 = Twitter), content (1 = braces removed, 2 = braces received, 3 = personal information about treatments/orthodontists/appointments, 0 = random/other), comedic value (0 = no, 1 = yes), presence/type of a selfie (0 = no, 1 = without visible braces, 2 = with visible braces). Content analysis of all included posts had been part of a pilot study and had been performed by two researchers separately and then jointly to find consensus [15]. In addition to this basic content analysis, screening all posts with regard to the presence of a selfie was part of the current investigation. In order to answer the core question about social perception of orthodontic-related posts, 42 first-year dental-school students (32 female, 10 male) aged between 18 and 33 years (M = 22.33, SD = 3.82) were asked to rate posters’ emotional states at the time of posting using the SAM-Tool modified to measure an observer’s perspective. Note that the exact age of the SNS users who posted the analyzed data could not be determined due to privacy policy reasons of the SNS. After screening all posts, the authors made the assumption, that the majority of SNS users were adolescents and young adults. The Ethics Committee of the University Hospital of Cologne approved this study (#19-1084_1). This approval was related to the students’ involvement with regard to coding and informed consent of these students was mandatory. The posts themselves were publicly available with no ethical approval needed.

**Fig. 2:**
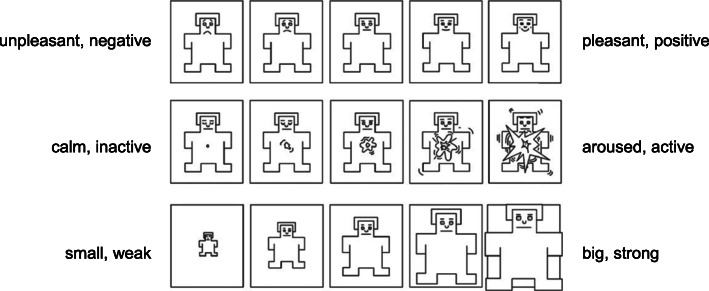
The five-point scale of Self-Assessment Manikins (SAM) [14] with the three emotional dimensions pleasure, arousal and dominance

All SAM assessments were collected using 5-point scales with perceived *pleasure* ranging from “unpleasant, negative” (=1) to “pleasant, positive” (=5), *arousal* from “calm, inactive” (=1) to “aroused, active” (=5) and *dominance* from “small, weak” (=1) to “big, strong” (=5; Fig. 2). Each post was rated by 9 to 13 raters (M = 10.50, SD = 0.88) with each rater evaluating printed batches containing between 99 and 101 posts (M = 100.26, SD = 0.86), blinded with regard to the corresponding SNS, leading to a total of 4211 poster-rater observations pertaining to the three SAMs dimensions. The data was organized in a “long format” with each row of data representing one poster-rater observation with the three SAMs dimensions serving as continuous dependent variables and the categorized information on the posts serving as independent variables.

First, a series of linear mixed regression models on the three key dependent variables was conducted using restricted maximum likelihood prediction and including random intercepts for both posters and raters. For each emotional dimension of perceived emotional states, main effects of platform, content, comedic value and presence/type of a selfie were accounted for. This procedure allowed the authors to estimate marginal means for the different values of any given predictor while simultaneously controlling for the influence of all other predictors. The estimated marginal means for the different values of each predictor were compared pairwise using Bonferroni adjustment for multiple comparisons (see (1) in results).

As a second approach to analyzing the data, a two-step cluster analysis was conducted to identify discrete clusters of emotional states as specific combinations of values on the three SAMs dimensions. These clusters were used as dependent variables in a mixed multinomial regression model using the same predictors as before and again including random intercepts for posters and raters (see (2) in results). All statistical analyses were performed with SPSS statistical package, version 27 (IBM). A *p*-value of less than 0.05 was considered to indicate statistical significance, while adjustment for multiple testing was performed as mentioned above.

## Results

### (1) Dimensions of perceived emotional states

**Fig. 3:**
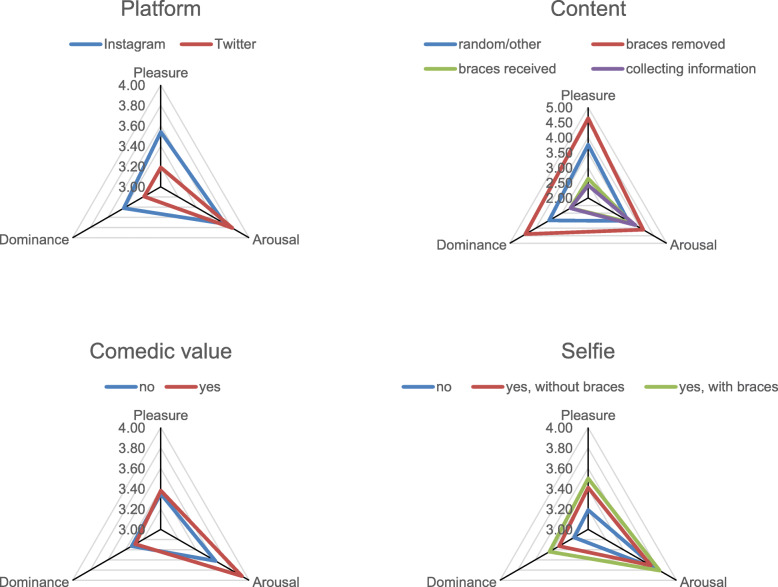
Estimated marginal means of posters’ perceived pleasure, arousal and dominance as a function of categorial predictor values

The tests of the fixed effects of the categorial predictors regarding all three dependent variables are presented in Table 1, the Bonferroni-adjusted multiple comparisons of the estimated marginal means are presented in Table 2 and visualized in Fig. 3.

**Table 1:** Fixed effects of categorial predictors on perceptions of posters’ pleasure, arousal and dominance

	DV: Pleasure		DV: Arousal		DV: Dominance	
Predictor	F	*p*	F	*p*	F	*p*
Intercept	2981.60	.00	5293.48	.00	3682.08	.00
**Platform**	7.69	.01	0.84	.36	5.17	.02
**Content**	123.13	.00	16.46	.00	121.72	.00
**Comedy**	0.13	.72	25.54	.00	0.42	.52
**Selfie**	3.18	.04	0.71	.49	4.33	.01

**Table 2:** Multiple comparisons of estimated marginal means of perceptions of posters’ pleasure, arousal and dominance between different values of categorial predictors

		Pleasure			Arousal			Dominance		
Predictor	Category	EMM	Sig. diff.	SE	EMM	Sig. diff.	SE	EMM	Sig. diff.	SE
**Platform**	*Instagram (1)*	3.54	(2)	0.08	3.73	n. s.	0.06	3.42	(2)	0.07
	*Twitter (2)*	3.19	(1)	0.10	3.81	n. s.	0.08	3.19	(1)	0.08
**Content**	*random/other (0)*	3.76	(1),(2),(3)	0.08	3.52	(1),(3)	0.06	3.49	(1),(2),(3)	0.07
	*braces removed (1)*	4.64	(0),(2),(3)	0.10	4.10	(0),(2),(3)	0.08	4.40	(0),(2),(3)	0.08
	*braces received (2)*	2.64	(0),(1)	0.11	3.64	1	0.08	2.67	(0),(1)	0.09
	*personal orthodontic-related information (3)*	2.42	(0),(1)	0.11	3.80	(0),(1)	0.08	2.68	(0),(1)	0.09
**Comedic value**	*No (0)*	3.35	n. s.	0.05	3.62	(1)	0.05	3.33	n. s.	0.05
	*Yes (1)*	3.38	n. s.	0.09	3.92	(0)	0.07	3.29	n. s.	0.08
**Selfie**	*No (0)*	3.19	(2)	0.07	3.78	n. s.	0.06	3.16	(2)	0.06
	*yes, without braces (1)*	3.41	n. s.	0.12	3.71	n. s.	0.09	3.33	n. s.	0.10
	*yes, with braces (2)*	3.50	(0)	0.09	3.81	n. s.	0.07	3.44	(0)	0.08

*Note:* Estimated marginal means (EMM) and standard errors (SE) are reported as predicted by the regression model. Significant differences (Sig. diff.) between a given category and other categories within the same categorial predictor are denoted with the respective comparison category identifiers in brackets (*p* < .05, Bonferroni-adjusted for multiple comparisons)

#### Social media platform

The social media platform was significantly associated with the perceived pleasure of the poster (F (1, 394.13) = 7.69, *p* < .01; Table 1) with Instagram posts receiving higher ratings (EMM = 3.54, SE = 0.08) than posts on Twitter (EMM = 3.19, SE = 0.10; Table 2). The same pattern was true with regard to perceived dominance (F (1, 393.67) = 5.17, *p* < .05; Table 1) with posts on Instagram obtaining higher ratings on this dimension (EMM = 3.42, SE = 0.07) than posts on Twitter (EMM = 3.19, SE = 0.08; Table 2). However, there was no significant effect on ratings of arousal (F (1, 394.58) = 0.84, *p* = .36; Table 1).

#### Content

Turning to the posts’ content, we also observed a significant effect on ratings of pleasure (F (3, 394.13) = 123.13, *p* < .001; Table 1). Post-hoc analysis revealed that getting braces removed was associated with higher perceived pleasure (EMM = 4.64, SE = 0.10; Table 2) than any other content category. Likewise, there was an effect on arousal (F (3, 392.70) = 16.46, p < .001) and dominance (F (3, 393.19) = 121.72, p < .001; Table 1) revealing the same pattern: Posts about getting braces removed evoked higher attributions of arousal (EMM = 4.10, SE = 0.08) and dominance (EMM = 4.40, SE = 0.08; Table 2) than posts pertaining to any other category. On the other hand, the emotions relating to posts about receiving braces (pleasure: EMM = 2.64, SE = 0.11; arousal: EMM = 3.64, SE = 0.08; dominance: EMM = 2.67, SE = 0.09) and personal orthodontic-related information (pleasure: EMM = 2.42, SE = 0.11; arousal: EMM = 3.80, SE = 0.08; dominance: EMM = 2.68, SE = 0.09) did not significantly differ in any of the three dimensions but generally showed lower values than any of the other categories, including random/other posts (pleasure: EMM = 3.76, SE = 0.08; arousal: EMM = 3.52, SE = 0.06; dominance: EMM = 3.49, SE = 0.07; Table 2). Note that on the arousal dimension, the difference between receiving braces and random/other was not significant.

#### Comedic value

Regarding the comedic value of social media posts, there was no significant effect on pleasure (F (1, 393.24) = 0.13, *p* = .72*),* or dominance (F (1, 391.96) = 0.42, *p* = .52), whereas we found an effect on arousal (F (1, 391.72) = 25.54, *p* < .001; Table 1). In particular, post containing humor called forth stronger attributions of posters’ arousal (EMM = 3.92, SE = 0.07) than non-humorous posts (EMM = 3.62, SE = 0.05; Table 2).

#### Presence or absence of a selfie

Whether the post contained a selfie had an effect on perceived pleasure (F (2, 392.87) = 3.18, *p* < .05) and dominance (F (2, 391.52) = 4.33, p < .05; Table 1), but not on arousal (F (2, 391.79) = 0.71, *p* = .49; Table 1). Post-hoc analyses showed that selfies with visible braces obtained significantly higher ratings of pleasure (EMM = 3.50, SE = 0.09) and dominance (EMM = 3.44, SE = 0.08) than posts containing no selfie at all (pleasure: EMM = 3.19, SE = 0.07; dominance: EMM = 3.16, SE = 0.06; Table 2). Yet, posts with visible braces were not rated significantly higher on these dimensions than posts without visible braces (pleasure: EMM = 3.41, SE = 0.12; dominance: EMM = 3.33, SE = 0.10; Table 2). As an additional analysis, all posts containing a selfie were categorized with regard to posters’ presumed sex (1 = female, 2 = male). However, when entered into our regression models, this predictor had no significant effect on either perceived arousal (F (1, 217.67) = 0.79, *p* = .38) or dominance (F (1, 218.79) = 0.59, *p* = .44) and only achieved marginal significance with regard to perceived valence (F (1, 218.43) = 3.32, *p* = .07), indicating that female posters (EMM = 3.62, SE = 0.16) tended to be perceived as feeling slightly more positive than male posters (EMM = 3.43, SE = 0.18), albeit not within a conventional level of significance.

### (2) Clusters of discrete emotions

**Fig. 4:**
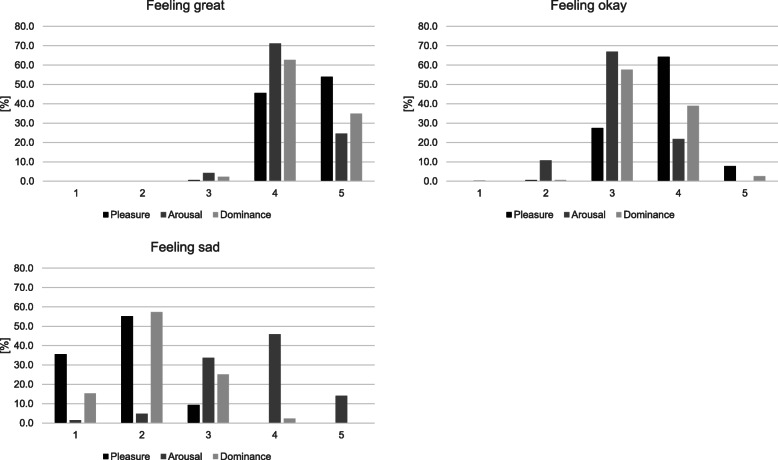
Relative distributions for perceived pleasure, arousal and dominance within clustered emotional states

The two-step cluster analysis identified three clusters of distinct emotional states (Table 3, Fig. 4). The largest cluster (43.9%) contained posts predominantly representing intermediate levels of pleasure (M = 3.79, SD = 0.57), arousal (M = 3.10, SD = 0.57) and dominance (M = 3.44, SD = 0.56) and was labeled as “feeling okay”. The second largest cluster (32.9%) was characterized by high pleasure (M = 4.53, SD = 0.51), high arousal (M = 4.20, SD = 0.50) and high dominance (M = 4.33, SD = 0.52) and was therefore labeled as “feeling great”. The last cluster (23.2%) contained posts representing low perceived pleasure (M = 1.74, SD = 0.62), intermediate arousal (M = 3.66, SD = 0.83) and low dominance (M = 2.14, SD = 0.69) and was labeled as “feeling sad”.

**Table 3:** Frequencies of clustered emotional states with means and standard deviations for posters’ perceived pleasure, arousal and dominance

		Pleasure		Arousal		Dominance	
Cluster	Frequency	M	SD	M	SD	M	SD
*Feeling great*	1385 (32.9%)	4.53	0.51	4.20	0.50	4.33	0.52
*Feeling okay*	1850 (43.9%)	3.79	0.57	3.10	0.57	3.44	0.56
*Feeling sad*	976 (23.2%)	1.74	0.62	3.66	0.83	2.14	0.69

The multinomial regression model predicted 82.6% of the cases correctly (feeling great: 79.1%; feeling okay: 81.6%; feeling sad: 89.7%). With “feeling okay” used as reference category for the dependent variable, we were now able to identify which variables predicted “feeling great” or “feeling sad” rather than “feeling okay”, respectively (Table 4).

**Table 4:** Fixed effects of categorial predictors on clustered emotional states

Cluster (ref.: *feeling okay*)			t	p	Exp(B)	95% CI for Exp(B)	
	Predictor					Lower	Upper
***feeling great***	Intercept		−3.83	.00	0.24	0.11	0.49
	**Platform** (ref.: *Instagram*)	*Twitter*	−0.57	.57	0.79	0.35	1.77
	**Content** (ref.: *random/other*)	*braces removed*	8.04	.00	18.39	9.04	37.42
		*braces received*	−2.19	.03	0.38	0.16	0.90
		*personal orthodontic-related information*	−1.26	.21	0.53	0.20	1.42
	**Comedic Value** (ref.: *no*)	*yes*	0.57	.57	1.16	0.70	1.91
	**Selfie** (ref.: *no*)	*yes, with braces*	1.86	.06	1.95	0.96	3.96
		*yes, without braces*	1.14	.26	1.66	0.69	4.01
***feeling sad***	Intercept		−4.91	.00	0.05	0.02	0.17
	**Platform** (ref.: *Instagram*)	*Twitter*	2.35	.02	4.54	1.28	16.09
	**Content** (ref.: *random/other*)	*braces removed*	−2.42	.02	0.05	0.01	0.57
		*braces received*	7.00	.00	31.92	12.10	84.23
		*personal orthodontic-related information*	6.49	.00	33.77	11.67	97.72
	**Comedic Value** (ref.: *no*)	*yes*	−2.11	.04	0.37	0.15	0.93
	**Selfie** (ref.: *no*)	*yes, without braces*	−0.19	.85	0.85	0.17	4.40
		*yes, with braces*	−1.72	.09	0.33	0.09	1.16

*Note:* Dependent variable (cluster) reference category: *feeling okay*. Reference category for each predictor in brackets

#### Feeling great

While neither the social media platform (*p* = .57) nor the comedic value of a post (p = .57) increased the probability of a post to count among the “feeling great” cluster rather than the “feeling okay” cluster, the different types of content had distinct effects: compared with the reference category (“random/other”), personal orthodontic-related information was not associated with “feeling great” (*p* = .21), but receiving braces predicted a significant drop in the odds of “feeling great” rather than “feeling okay” (Exp(B) = 0.38, *p* < .05), whereas getting braces removed predicted a considerable increase in the odds of “feeling great” (Exp(B) = 18.39, *p* <. 001). The presence or absence and type of a selfie also appeared to have a slight effect: Selfies with visible braces had marginally higher odds of being perceived as “feeling great” than posts containing no selfie (Exp(B) = 1.95, *p* = .06), yet not statistically significant (Table 4).

#### Feeling sad

Posts on Twitter were significantly more likely to be clustered as “feeling sad” rather than “feeling okay” than posts on Instagram (Exp(B) = 4.54, *p* < .05). With regard to the posts’ content (as compared to “random/other”), posts about getting braces removed had a considerably lower chance of being associated with “feeling sad” (Exp(B) = 0.05, p < .05), whereas posts pertaining to either receiving braces (Exp(B) = 31.92, *p* < .001) or personal orthodontic-related information (Exp(B) = 33.77, *p* < .001) yielded significantly higher odds to count among the “feeling sad” cluster. Furthermore, posts containing comedic elements were less frequently predicted to count among the “feeling sad” cluster (Exp(B) = 0.37, p < .05). Finally, neither posts containing a selfie with (*p* = .09) nor without visible braces (*p* = .85) had a conventionally significant effect on the odds of “feeling sad” rather than “feeling okay” as compared to posts containing no selfie at all, with the former however suggesting a marginally significant decrease (Exp(B) = 0.33; Table 4).

## Discussion

To the authors’ knowledge, this is the first study that investigated the social perception of orthodontic-related posts on Twitter and Instagram by young adults. Using the Self-Assessment Manikin-Tool, three dimensions – pleasure, arousal and dominance – of the posters’ assumed emotional states could be evaluated at the time of the relevant orthodontic-related posts [14]. Although two-dimensional pleasure-arousal models are commonly used in relevant literature as well [23, 24], the authors decided to use the three-dimensional pleasure-arousal-dominance model according to Bradley and Lang, like it has been frequently part of psychological studies throughout the past years [14, 25–27], in order to get an even broader view. This research method might be used for future and similar studies in order to explore posts on SNS in the dental context.

Previously assigned content categories were significantly related to specific dimensions of the emotional state of the person who posted and a reference to discrete emotions was established through cluster analysis. The emotional states of posters writing about getting their braces removed were on average judged to be more positive, more excited and more dominant than the emotional states of posters writing about any other content. Similarly, posts that were assigned to the category ‘braces removed’ were significantly more likely to be allocated in the ‘feeling great’ cluster. Discrete emotions like *happiness, joy* and *excitement* could be associated with this cluster [28–31]. On the other hand, posts about receiving braces or personal information about treatments/ orthodontists/ appointments were seen as indicative of comparatively negative, unexcited and submissive emotional states and were significantly more likely to be assigned to the ‘feeling sad’ cluster. *Anger*, *distress* and *fear* might be discrete emotions within this cluster [28–31]. This perception sounds alarming at first sight. Yet, several aspects have to be thought of in this context. Oral-health-related quality of life of patients might be impaired during the time of fixed appliance treatment and adjusts positively when active orthodontics end [32–38]. Orthodontic patients might suffer from temporary pain due to bracket and wire insertion, for example, which could explain feelings like *distress* or *anger* in the context of inserting orthodontic appliances. Patient satisfaction during orthodontic treatment might be altered because of potential braces-induced impairments, but has been proven to be high after finishing active orthodontic treatment [39], just like oral-health-related quality of life as mentioned above. Researchers like Paes da Silva et al. found out, that patients with inserted braces were bothered by concerns regarding the appearance of their teeth and the impact on social interactions, among others [32]. Furthermore, patients might *fear* specific orthodontic appointments like bracket insertion because of their peers’ personal stories about them – which, sometimes, might not be a truthful version of reality and potentially exaggerated. Also, some patients might complain about long waiting times at the office. It has been discussed in relevant literature that whenever orthodontic-related posts were rated as being *negative*, there was a large number of posts containing information about orthodontists and orthodontic appointments within [15]. Orthodontists should regard the results of this research as a sign to work harder towards taking their patients’ fears in relation to appointments and procedures as serious as possible.

Contradictory and surprising at first sight, the results revealed, that SNS users who showed their braces within a selfie-post were on average judged as feeling more positive and stronger than those who did not post a selfie. Such selfie-posts were more likely to be assigned to the ‘feeling great’ cluster with the previously mentioned discrete emotions *joy* and *happiness* potentially connected to it. Although posters were perceived to have rather negative feelings about the insertion of an orthodontic appliance, they seemed to present their braces in a much better light whenever they included a selfie within the corresponding post. These findings might reveal a modern and SNS-related coping mechanism of teenagers and young adults during the phase of active orthodontic treatment: Young patients might want to show themselves happy and comfortable with their braces in public. Apparently, seemingly self-confident users spread positive attitudes with regard to the perception of orthodontics through smiling selfies with visible braces. By receiving positive reactions and rewards in the form of ‘likes’, such users might be strengthened in their way of thinking about themselves. In modern times of highly frequented social media use by young adults, this might be seen as a new way to cope with potentially compromised situations due to a specific therapy, e.g. fixed appliances. Although patients with braces might be concerned about their social interactions and their looks [32], some seem to reverse these sorrows into a positive way of coping through social interactions on SNS. Since high esthetics and facial appearance play a crucial role for self-esteem and social interactions nowadays [5–8], many adolescents wish for an improvement of their malocclusion, regardless of their normative orthodontic treatment need [40], potentially to feel more confident and approved by their peers. In this context, one has to keep in mind that specific dentofacial traits have been proven to be associated with bullying in adolescents and young adults [41, 42]. Thus, the correction of such dentofacial malformations in the course of an orthodontic treatment meets the current needs of some adolescents, as mentioned above, with the consequence of proudly presenting their appliances on SNS. In line with these thoughts, Patel et al. found out that British school children did not make social judgements solely because of visible fixed orthodontic appliances. Orthodontic treatment as such has become rather normal for adolescents and might not be the cause for teasing as it has been in former times [43, 44]. On the other hand, one should not neglect the potential harm revolving around the anonymity of the internet and of SNS. As selfie-taking might be associated with specific personality traits [45] and as approval and belonging are among the key reasons for their use [12, 13], there might also be another side of the story: While it might be rewarding and supportive to receive positive feedback after posting a selfie with visible braces, it might be equally disturbing and even devastating for young patients to experience negative reactions by anonymous users. In this context, research about bullying related to dentofacial appearance and orthodontics on SNS is still sparse [19]. Orthodontists should be aware of the social-media-related phenomenon mentioned above and the potential coping mechanisms of young patients. They might try to encourage positive feelings about necessary orthodontic appliances and emphasize their apparent social acceptance nowadays.

In addition to that, another major research question of this study had been the potential SNS-related difference in the investigated social perception of orthodontic-related posts. While posters on Instagram and Twitter were on average perceived as equally excited or unexcited, posters on Instagram were perceived as feeling more positive and stronger than their peers on Twitter. Orthodontic-related posts on the text-based SNS Twitter were significantly more likely to be clustered as “feeling sad” rather than “feeling okay” than posts on the photo-based SNS Instagram. These orthodontics-focused findings are reinforced by the general findings of Jeri-Yabar et al., who stated that depressive symptoms in relation to excessive use of SNS were found to be more frequent among Twitter users than among users of other SNS [9]. As it has been discussed before, users might feel more free to post about complains and fears in a text-based form than they do in combination with posting a picture – potentially a much more personal selfie [15]. Interestingly enough, our previously mentioned results regarding the social perception of specific orthodontic-related *content* can be analyzed independently of such crucial SNS-related differences because of our methodological approach.

A major limitation of this study lies within its very nature. Although social media research is wished for in the field of orthodontics [46], it is hard to aim for generalizability in this field of research. Especially adolescents and young adults – representing the typical orthodontic patient as well – frequently use SNS in order to communicate and connect with others. They either do it in *private* by limiting information to their peers or other users they know or post about their feelings and thoughts in *public*. In general, researchers have easy access to publicly available posts on SNS, like in the current study, and though this can be regarded as a major research advantage, it might be also seen as an important drawback of social media research: Users who post on SNS in public might not represent *all* current and/or future orthodontic patients. Furthermore, it is worth mentioning that public posts within the *virtual reality* might not necessarily correlate with one’s *real* feelings and emotions; yet, they might be guiding young people’s actions. In addition to that, first-year dental students were part of this research about the social perception of orthodontic-related posts on SNS. These subjects might perceive the emotions of users differently than other members of our scientific community and – maybe more importantly – the users themselves. Moreover, age- and socio-economic-related factors were not accounted for. Thus, the presented results can only be interpreted as a hint to potential social perception of orthodontic-related social media use, but cannot ensure generalizability. Yet, it is highly important to ensure that patients’ perspectives are investigated in the best possible way in order to understand their thoughts, emotions and behaviors.

## Conclusion

This study gives insights about the social perception of orthodontic-related posts on Twitter and Instagram by young adults. Users’ posts were perceived highly differential on both SNS, being that posts on Twitter were significantly associated with supposedly negative emotions of the users. Regardless of the SNS, this research revealed content-based differences as well: Receiving orthodontic appliances was significantly associated with rather negative emotions of the person who posted, while users who posted about the removal of such braces were perceived as significantly more joyful. Interestingly, young adults perceived users whose posts contained selfies *with* visible braces as significantly more positive and stronger in comparison to users who did not post a picture of themselves. Thus, a novel and social media-related way of coping with a supposedly temporary impairment like fixed orthodontic appliances emerged and should be looked at in detail in future studies, especially in modern times of frequent social media use among young adults.

## Supplementary Information

**Additional file 1.** Reviewer reports and responses

## Data Availability

The data underlying this article cannot be shared publicly for no clear reason due to privacy issues of individuals that participated in the study. Yet, the data will be shared on reasonable request to the corresponding author.
