# Supplementary material for: Looking good but tweeting bad? The social perception of orthodontic-related posts on Twitter and Instagram
Source: Head Face Med. 2022 Feb 17;18:6. doi: 10.1186/s13005-021-00302-1 (PMC8851782; doi:10.1186/s13005-021-00302-1)
Supplement: Supplementary file 1 — Additional file 1. Reviewer reports and responses [file 13005_2021_302_MOESM1_ESM.docx]

Dear Professor Dr. Stamm,

Thank you very much for your E-mail and the information regarding our submission. We are pleased to hear that the reviewers find our study to be interesting and well written. On the other hand, we take their critical remarks very seriously and hope that our revision meets their standards/the standards of your journal.

Please find our detailed answers to each of the reviewers’ remarks as follows, highlighted in yellow. We revised our manuscript thoroughly and according to the reviewers’ recommendations and highlighted parts of our manuscript in grey, whenever we adapted the text.

We would be very honored, if our research project was published in the Head and Face Medicine and are looking forward to the further publication (and revision) process.

Sincerely,

Isabelle Graf

___

**Reviewer reports and responses:**

**Reviewer #1**: Language review - very good

Thank you very much.

**Reviewer #2**: As a fellow proponent of the use of social media to uncover societal trends towards health and healthcare, I have found this article to be quite encouraging. After reading the manuscript I have raised several questions and comments.

We appreciate your valuable comments and thank you for the time you have put into your review. We hope to meet your expectations by revising our manuscript according to your remarks – as much as possible.

**Abstract**

From reading the abstract I was initially led to believe that the research is exploring the perception of young people who wear braces and post about it on SM. This really isn't clear and I was left confused. This needs re-writing for clarification, may I suggest the following -. The aim of this research was to explore the social perception of orthodontic-related posts on Twitter and Instagram posted by adolescents and read by young people.

Thank you for your remark and your suggestion. Due to privacy policy restrictions of Instagram and Twitter, we cannot be sure about the age of the social media users. We assume that most users were adolescents because of the pictures (e.g. selfies) they posted. Thus, we only know the age of those young people who rated the posts, but not the exact age of the persons who posted our orthodontic-related posts. We tried to clarify this within the methods- section.

**Introduction**

Because of the aforementioned it is not clear what the ethical approval was sought for. SM posts are posted within a public domain and therefore consent to use is not required, this should be mentioned in the research. Postee's would have this as a disclaimer for use in the app's T&C'S. If ethical approval was sought for the students' involvement in coding the posts then this needs to be made clearer.

We clarified this point within the methods- section.

Avoid writing in the first person.

We revised the whole manuscript regarding the use of the first person. Thank you for mentioning.

I'm unsure as to why there is a need to investigate the social perception of orthodontic posts, there needs to be stronger justification for the research.

Thank you for letting us know, that this crucial aspect needed to be emphasized. In general, social media has a huge potential to influence young people, especially young people within public health context (and in specific: within orthodontic context). Our behavior and our “perceived reality” can be regarded as being highly driven by our *perception* of others, for instance, which is a key aspect of Bandura’s Social Learning Theory/ Social Cognitive Theory (1977). We added some thoughts about this and the Thomas Theorem into the introduction-section in order to underline the importance of social perception.

**Methods**

How and why were key words chosen?

We searched both SNS for orthodontic-related hashtags/keywords and found that the keywords used within our study would reflect most of the orthodontic-related posts. Please note that the current study is a continuation of a pilot study which had been published in the American Journal of Orthodontics and Dentofacial Orthopedics (Graf I, Gerwing H, Hoefer K, Ehlebracht D, Christ H, Braumann B. Social media and orthodontics: A mixed-methods analysis of orthodontic-related posts on Twitter and Instagram. Am J Orthod Dentofacial Orthop. 2020;158(2):221-8). Needless to say, only new data according to our additional and ongoing analyses are presented within the current manuscript.

Why Instagram and Twitter. These platforms are quite different and used for different purposes, it is of no surprise that you found significant differences between the posts.

We chose Twitter and Instagram because both SNS are frequently used by young people, who are often orthodontic patients as well. We mentioned this aspect within our manuscript. Few studies about the SNS Twitter already exist in the orthodontic context, while there is no relevant study about the SNS Instagram currently – to our knowledge. Both SNS have in common, that they are used for real-time communication although their nature is quite different, as you mentioned as well. We agree with you, that it is not surprising to find SNS-related differences in the perceived emotional state of the users between both SNS; yet, this had to be verified because there had been no orthodontic-related data about this phenomenon. In this context, we would like to stress that we believe such results to be interesting for the reader/for orthodontists/for dentists, because in order to fully understand our patients’ needs and thoughts, we should also look at what they do/write in their “natural environment” and how their actions in this environment are perceived by others – virtual reality (and with it SNS) belongs to young peoples natural environment. Thus, it is worth looking at.

Another SNS of interest could have been Facebook. Nevertheless, we stuck to the above-mentioned SNS; the search strategy (keywords/hashtags) would have been rather different between Instagram/Twitter and Facebook. Including Facebook would have led to even more data which would have potentially led to more confusion within the results section. We hope that you agree with us, that it is no limitation of our study, that we focused on Twitter and Instagram, especially holding the fact that most studies within this context focus on only one SNS.

Who collected the data? Who analysed the posts regarding its inclusion and exclusion criteria? What was the inclusion criteria?

One researcher collected the data under supervision of the principle investigator. Data was then analyzed according to Mayring’s content analysis by two researchers separately and then by both together to find consensus. By following all steps of the above-mentioned content analysis, five main categories were initially found within the content of all orthodontic-related posts. Please note that 361 posts out of the 401 posts which were the data base of the current study had been part of the above-mentioned publication in the American Journal of Orthodontics and Dentofacial Orthopedics. 40 posts were not excluded in the course of this study as they had been before: These were mostly comedic posts made by the snapchat tool which we found to be of interest within the current study. We included all posts with the relevant keywords as described (=inclusion criteria), but excluded quite a few according to our exclusion criteria (n=148 excluded out of a total of 549 orthodontic-related posts). In order to clarify this, we added a flow chart of included and excluded posts and added more information about gathering and analyzing posts in the methods-section.

This research is exploring the perception of young adults who read the posts of people who post about their braces. Where is the link to adolescents and SM posts?

Thank you for this thoughtful remark. As we stated above and added to our manuscript, we strongly assume that the majority of SNS users within our study were adolescents and young people. Unfortunately, we cannot be fully sure about this.

How did you select the posts, was the inclusion criteria age to include adolescents, this is not specified.

As mentioned before, we were not able to find out how old the SNS users had been. Thus, this had been no inclusion criteria. Our inclusion criteria involved the keywords “braces”, “orthodontics” and “orthodontist” within a thirty-day-period- in accordance with relevant literature (e.g. Henzell, Knight, Morgaine et al.) .

Number of posts obtained is a result and needs to be moved to the results section.

Thank you for this recommendation. Because we already published the “base data” and the corresponding content analysis according to Mayring as mentioned above, we did not believe that data collection is a result of this specific study, but rather of the pilot study that had been published. We think that it would be confusing and somehow duplicate to present this data in the results-section and hope that you agree with us after reading our answers and comments. Nevertheless, we added a flow chart to our manuscript in order to clarify the search/included and excluded posts.

**Results**

This section is a bit confusing rather than having them as appendices presenting the main results in the tables/graphs would be easier to read and more pleasing on the eye.

Thank you for this comment. We somewhat agree with you; our results might be a bit confusing due to the rather uncommon statistical analyses within the orthodontic/dental context. Yet, we would strongly suggest them to be presented as currently because it is rather ‘state-of-the-art’ in the field of sociology and social psychology, where the methodological approaches to analyzing the data are based in. Furthermore, we might have been mistaken, but our figures and tables should not be seen as appendices, but should be rather presented next to the corresponding text parts, at least in the final version of the manuscript. We believe that this can be realized in a more pleasing way in the course of formatting (in case of publication). We hope that you agree with us.

Examples of the tweets/posts should be included in the results section or appendices.

This is a very good remark and we thought about this a lot. Because of privacy policy and possibly copyright restrictions we were not allowed to present posts containing pictures as examples of our data. We discussed this with the legal department of our university. In addition to that, we did not want to present a repetition of our previously published and above-mentioned work in the American Journal of Orthodontics and Dentofacial Orthopedics and mention examples of tweets or text-based posts but rather concentrate on the social perception of such posts by young adults. We hope that you agree with us. If not, we will of course willingly present examples of text-based tweets within the specific content categories which differ from the previously presented.

**Discussion**

Write in the third person - To the 'author's' knowledge as opposed to 'our' and avoid using 'we' - but this is just minor nit picking.

We adapted this within the discussion-section. Thank you for your remark.

**Conclusion**

A nice summary of your findings.

Thank you very much!

Overall the article is interesting although I'm unsure of its impact. You could perhaps strengthen this by suggesting that this research method could be used for similar studies that look at SM posts and perceptions.

We added this thought in the discussion section.

**Reviewer #3**: The idea of examining the social perception of patients under orthodontic treatment is generally interesting.

Thank you very much! We highly appreciate your positive attitude towards our research project and the corresponding manuscript and hope to meet your expectations by revising our manuscript according to your remarks.

As mentioned in the Background, the social networking sites play an immense role in the everyday life of young adults, who at the same time make up the majority of the patient clientele in orthodontics. The SNS can therefore be seen as a huge data set of emotional highlights in the everyday life of the users. For an understandable overview, the criteria for including suitable posts must be set narrowly, as done in this paper. The preselection limits the content to the most drastic changes during a typical orthodontic treatment.

Thank you for this reflection.

The methodology can only be an approximation, which is why I welcome the choice of the SAM-Tool and its expansion to 3 dimensions.The comparison of a more image-based and a text-based site is also interesting as we can see on the results.

For me the textual presentations of the results are a bit confusing and not intuitively understandable. Nevertheless, the key messages are made clear.

Thank you. We somewhat agree with you. Our data analyses might be quite complex due to the vast amount of data and complex statistical procedures needed to test the significance of the results. Yet, we tried to present the most important results both in a visual and textual way, so that readers might be able to get a broad view of all data- without leaving important information out.

The derived clusters help to understand this further.

The discussion is varied, differentiated and provides comprehensible attempts to explain the found results. In particular, it is important to point out that some patients may be emotionally preoccupied with exaggerated posts from their peers, especially for orthodontists, who have no experience with SNS themselves and who therefore cannot necessarily understand these patient's concerns.

The weaknesses of the study design are also correctly highlighted here and coincide with the objections that I would have to point out. Of course it is not possible to derive generalizations from this study. Besides having a selective group of future professionals rating the posts may leads to a perception bias. In addition, it is worth to be mentioned, that public emotional posts do not necessarily correlate with one person's real feelings. Often there is a large discrepancy between the virtual media world and reality.

Thank you for these remarks. We added these thoughts to our discussion-section.

The summary provides a conclusive interpretation of why selfies with brackets may serve as a suitable confirmation strategy for bracket insertion, which is otherwise associated with rather negative feelings.

Again, thank you for your time and effort while revising our manuscript.
